# Augmented reality navigation method based on image segmentation and sensor tracking registration technology

**DOI:** 10.1038/s41598-024-65204-z

**Published:** 2024-07-03

**Authors:** Xiaoying Zhang, Yonggang Zhu, Lumin Chen, Peng Duan, Meijuan Zhou

**Affiliations:** College of Mechanical Engineering, Zhengzhou University of Science and Technology, Zhengzhou, 450064 China

**Keywords:** Image segmentation, Sensors, Augment reality, Track registration, Mathematics and computing, Physics

## Abstract

With the rapid development of modern science and technology, navigation technology provides great convenience for people's life, but the problem of inaccurate localization in complex environments has always been a challenge that navigation technology needs to be solved urgently. To address this challenge, this paper proposes an augmented reality navigation method that combines image segmentation and multi-sensor fusion tracking registration. The method optimizes the image processing process through the GA-OTSU-Canny algorithm and combines high-precision multi-sensor information in order to achieve accurate tracking of positioning and guidance in complex environments. Experimental results show that the GA-OTSU-Canny algorithm has a faster image edge segmentation rate, and the fastest start speed is only 1.8 s, and the fastest intersection selection time is 1.2 s. The navigation system combining the image segmentation and sensor tracking and registration techniques has a highly efficient performance in real-world navigation, and its building recognition rates are all above 99%. The augmented reality navigation system not only improves the navigation accuracy in high-rise and urban canyon environments, but also significantly outperforms traditional navigation solutions in terms of navigation startup time and target building recognition accuracy. In summary, this research not only provides a new framework for the theoretical integration of image processing and multi-sensor data, but also brings innovative technical solutions for the development and application of practical navigation systems.

## Introduction

In today's context of urbanization and rapid development of information technology, accurate and fast navigation technology has become an indispensable helper for modern people to travel^1,2^. However, due to the tall buildings and signal interference in cities, traditional navigation tools such as GPS often lose their positioning accuracy in these urban environments, which not only causes navigation errors, but also seriously affects the user experience. In the face of this challenge, augmented reality (AR) technology is considered one of the key technologies to improve navigation accuracy and user experience due to its ability to seamlessly integrate virtual information with the real world^3,4^. In recent years, although various methods based on deep learning have made remarkable achievements in image processing and pattern recognition, the complexity and power consumption of these methods have become constraints in the face of resource-constrained augmented reality application scenarios^5^. To address this challenge, this research proposes an augmented reality navigation method that combines fused image segmentation techniques with virtual space transformation techniques. Unlike existing literature that mostly relies on resource-intensive computational models, this method aims to accomplish accurate image segmentation through GA-OTSU-Canny algorithms and combine sensor localization techniques to achieve a mobile augmented reality terminal-friendly and computationally efficient augmented reality navigation experience. Compared with the existing techniques, the method proposed in the paper not only theoretically proposes a new framework for integrating image processing and sensor data, but also experimentally verifies its significant effect on improving navigation accuracy and reducing startup time in practical applications. The innovativeness of the combined technique used in the paper is mainly reflected in the fact that the GA-OTSU-Canny algorithm can automatically select the optimal threshold value, making it more effective for globally consistent images, and thus better processing of target building images in navigation problems. In addition, by combining the data from various sensors such as GPS, gyroscope, accelerometer, etc., the real-time tracking and calibration of the device position in complex environments can be realized. The main contributions of this research are threefold. First, a novel navigation system combining image segmentation and sensor tracking and calibration techniques is proposed and realized, which effectively improves the navigation accuracy in complex environments. Second, the GA-OTSU-Canny algorithm enables the navigation software to recognize and process target building images more accurately, providing users with a more accurate navigation experience. Third, it provides a new idea and technical solution, which has a wide practical application value for intelligent traveling mode. In conclusion, this study not only realizes obvious innovations in technology, but also has a wide range of prospects in practical applications.

## Related works

With the development of the information technology industry, more and more technical personnel are committed to transforming virtual information into tools that contribute to human real life, and constantly opening up new fields. Digital Image Processing Technology (DIP-tech) is a promising fundamental algorithm for computer vision. Image segmentation algorithms, as an important category of DIP-tech, have certain practical value due to their ability to segment feature regions from other regions in an image. So it is often applied for research in different fields. Liu et al. devised a multi-criteria fuzzy clustering approach aimed at diminishing the influence of noise on the effectiveness of image segmentation. This approach incorporates dual fitness functions enriched with both local and non-local spatial data, and employs a strategic set optimization technique. The outcomes from their experiments indicate that the image segmentation methodology employed by this algorithm exhibits proficient noise attenuation capabilities alongside enhanced segmentation efficacy^6^. On another note, Belizario et al. introduced an automated technique for segmenting color images. This method initiates with a preliminary segmentation via superpixels, formulates three strategies for label propagation, and leverages color information within the images to distill features, culminating in an autonomous segmentation process. This approach not only reduces the duration required for segmenting images but also elevates the quality of the segmentation^7^. Furthermore, Shen et al. brought forth an innovative approach for partitioning daily traffic flows into distinct time slots through clustering. This technique involves formatting traffic flow data into a matrix, transforming it into images for segmentation, and subsequent analysis of these segments to determine the optimal duration of traffic signals. Simulations have confirmed the efficacy and applicability of this method in real-world settings^8^.

Besides the image segmentation technology (IS-tech), sensors continue to be a focal point in contemporary technological research, given their capacity to detect and convert information. The functional application of sensors is not limited by industry fields, so many industry technologies are optimized and developed based on sensors. Wu et al. designed artificial sensory neurons from a biological perspective. This neuron is connected in series with an optical sensor and an oscillating neuron, which can sense the visible light area of the outside world and automatically encode the perception information into electrical pulses through a neural network. This study has promoted the development of biology and intelligent sensors, laying the foundation for the construction of artificial vision systems^9^. Wang et al. raised a method to optimize multi wall carbon nanotube materials and applied it to aerogel flexible sensors^10^. This method aminated multi walled carbon nanotubes through dehydration condensation to form multi walled carbon nanotube composite fibers with chain ring structure. The composite fiber had a layered porous structure and certain mechanical elasticity, and could withstand a pressure resistance of 269.02 kPa. The flexible sensor made of this composite fiber could stably operate for 1000 cycles^11^. Zhou et al. designed a method for achieving high sensitivity capacitive pressure sensors. The method made relief on the hydrogel electrode, and adjusted the measurement coefficient of the pressure sensor according to the electrode structure and relief size. The data showed that the measurement coefficient of the capacitive pressure sensor with relief can reach up to 7.70 kPa, and the minimum sensing temperature could reach − 18 degrees Celsius. This sensor could be applied in the medical field to measure joint curvature, etc.^12^.

In summary, both IS-tech and sensor technology have played a significant role in the development of modern technology. Although many scholars have conducted in-depth exploration on the application of the two technologies in the field of navigation, the low efficiency of image segmentation methods and the large error of sensor technology have always been research challenges. This research proposes to combine the two technologies, using image segmentation algorithms to recognize and process building images, and sensor technology to optimize target localization, and applying the combination of the two technologies to augmented reality navigation, overlaying virtual images with real scenes in navigation to improve the recognition and localization of the real world.

## AR Navigation Method Based on IS-Tech and Sensor T & R-Tech

### Research on image processing methods in road segmentation

In augmented reality navigation, recognizing and segmenting target features in road images is a prerequisite for achieving target tracking, and image segmentation is the process of recognizing the information in images and marking them well for segmentation. It can be seen that image segmentation technology is an important support to realize augmented reality navigation. Image segmentation algorithms based on thresholding are mainly divided into OTSU algorithm, adaptive thresholding algorithm, etc. Among them, OTSU algorithm is widely used because it has the characteristics of small computation and high operation efficiency. This algorithm assumes that threshold $R$ can segment an image of size $A \times B$ into two parts: the target and background. Therefore, the number of target pixels $C_{0}$ is obtained from this, which is Eq. (1) ^13^.

1 $$C_{0} = \beta_{0} \times \left( {A \times B} \right)$$

$\beta_{0}$ in Eq. (1) represents the proportion of the target pixel to the overall pixel. From this, the number of background pixels $C_{1}$ can be inferred, i.e. Equation (2).

2 $$C_{1} = \beta_{1} \times \left( {A \times B} \right)$$

In Eq. (2), $\beta_{1}$ is the proportion of background pixels to the total pixels. Equation (3) can obtain the relationship between the number of target pixels and background pixels.

3 $$C_{0} + C_{1} = A \times B$$

By comparing the proportion of target pixels to background pixels, the total average grayscale $\varepsilon$ of the image can be obtained as Eq. (4).

4 $$\varepsilon = \beta_{0} \times \varepsilon_{0} + \beta_{1} \times \varepsilon_{1}$$

$\varepsilon_{0}$ in Eq. (4) is the average grayscale of the target pixel, and $\varepsilon_{1}$ is the average grayscale of the background pixel. The calculation formula for the known inter class variance $g$ is Eq. (5).

5 $$g = \beta_{0} \times \left( {\varepsilon_{0} - \varepsilon } \right)^{2} + \beta_{1} \times \left( {\varepsilon_{1} - \varepsilon } \right)^{2}$$

By combining Eq. (4) and Eq. (5), the simplified inter class variance formula can be derived as Eq. (6).

6 $$g = \beta_{0} \beta_{1} \times \left( {\varepsilon_{0} - \varepsilon_{1} } \right)^{2}$$

The inter class variance $g$ can be calculated from the above formula. When $g$ is the maximum, the variance between the background area and the target area is the maximum, and the threshold $R$ is the optimal value^14^.

**Figure 1:**
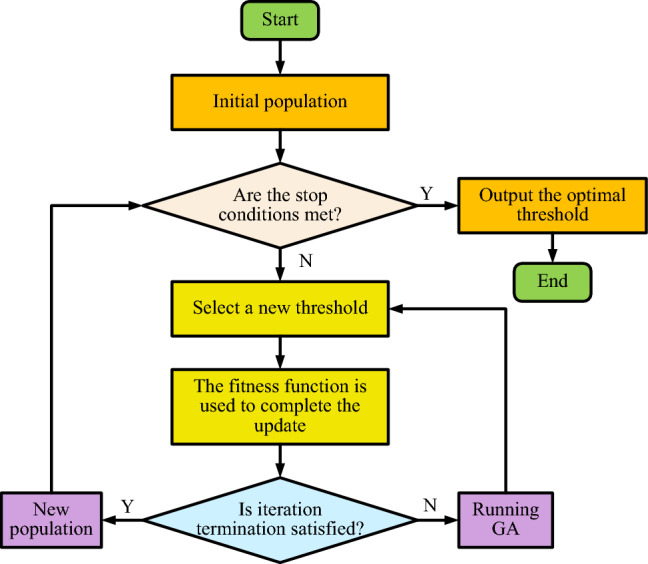
GA-OTSU operation flow chart.

In the process of road image analysis, OTSU algorithm is firstly used to segment the image to distinguish between road and non-road areas. Subsequently, Canny edge detection technology was used to further identify and determine the boundaries of these regions. In order to ensure that the non-road areas of the image are effectively removed and the road areas are clearly identified, the image is also closed. Considering the complexity of remote sensing images and the possibility that the same threshold may produce different segmentation effects in different geographical environments, the OTSU method optimized by Genetic Algorithm (GA) was introduced in this study, and the method was recorded as GA-OTSU. Ga-otsu automatically optimizes the threshold selection process of OTSU by utilizing the powerful search capability and fast computing capability of GA. In addition, GA-OTSU also improves the double threshold selection of Canny algorithm. By restricting the double threshold of Canny operator, the overall adaptability of Canny algorithm can be effectively improved, and the accuracy and efficiency of image processing can be improved. The operation flow chart of GA-OTSU is shown in Fig. 1.

In Fig. 1, population initialization is carried out first, and then whether the initialized population meets the stop condition is judged. If it meets the stop condition, the optimal solution threshold is output at this time and the operation of the algorithm is ended. If the stop condition is not met, a new threshold is set and a new population renewal is performed according to the fitness function. Next, it is determined whether the new result meets the condition of stopping iteration. If it does not meet the condition, the population is updated by GA algorithm, and the new threshold is selected. If yes, the iteration is stopped and a new population is obtained.

**Figure 2:**
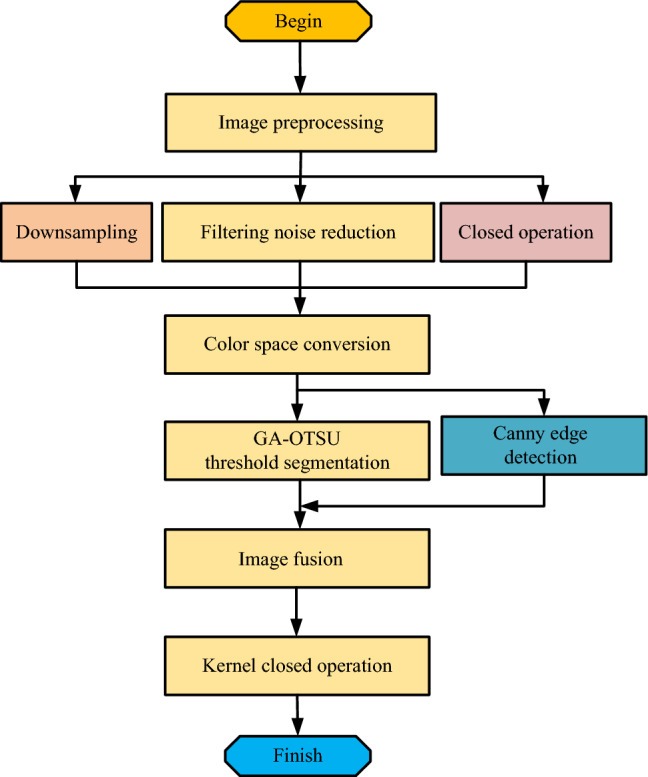
Road segmentation flow chart using image processing methods.

In GA-OTSU operation, the double threshold constraint means that points above the high threshold are treated as edge points, while points below the low threshold are treated as non-edge points. A point between a high threshold and a low threshold is determined to be an edge point by observing whether it is connected to an edge point above a high threshold. For a pixel with an intermediate gray value, its neighboring pixels within a specific target scale should be consistent or in the same homogeneous region. Based on this, the double threshold can be set by analyzing the statistical characteristics of pixel gray level in this region. First, the average gray value and standard deviation of pixels in the target scale range are calculated. Then, set the high threshold as a multiple of the mean plus the standard deviation, and the low threshold as a multiple of the mean minus the standard deviation. This strategy makes full use of the local statistical properties of pixels and can determine the high and low thresholds more accurately, thus significantly improving the effect and robustness of edge detection. Finally, the improved Canny operator edge detection algorithm combined with GA-OTSU is denoted as GA-OTSU-Canny, and the process of using GA-OTSU-Canny to complete road image segmentation is shown in Fig. 2.

Figure 2 shows the flow chart of road segmentation using image processing methods. Firstly, the image needs to be pre-processed, including down sampling, filtering and noise reduction, and closure operations. Then the color space of the image needs to be transformed to extract its color space channels. Then, the GA-OTSU method and Canny method are used for image thresholding and Canny edge extraction, respectively^15^. The images after threshold segmentation and edge extraction are fused, and the closed operation is performed on them, and then the processed images are obtained. This process can accurately identify and segment the target area of the road image and magnify and highlight it, and also eliminate the influence of non-road target area, which helps to realize the effect of sensor technology for its tracking and registration.

### Mobile AR navigation based on sensor technology

Target feature extraction of road images helps to implement tracking and registration techniques in augmented reality navigation. Augmented reality technology combines virtual information with real scenes and delivers the integrated information to the user so that the user can directly access the navigation information of real scenes^16^. Tracking and registration technology can locate the user's location and analyze the surrounding situation of his location to obtain the corresponding path and building information. The tracking and registration technology is implemented mainly through sensors for information transmission, and common sensors include GPS positioning sensors, acceleration sensors, gyroscope sensors, etc. The current sensors used by people for navigation through smartphones are usually a combination of these sensors. This study will implement path tracking registration based on image segmentation with multi-sensor technology, and finally fuse image segmentation technology with multi-sensor technology and apply it to augmented reality navigation system, aiming to improve the target object recognition accuracy and navigation accuracy of the navigation system.

**Figure 3:**
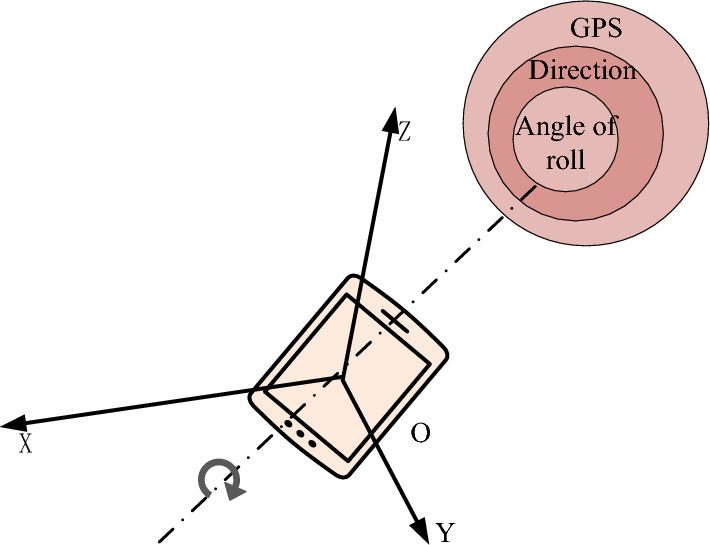
Sensor combinations in smartphones.

Figure 3 shows a common sensor combination diagram in smartphones. The method of tracking and registering this sensor combination is to establish a multi-layer structure, refine the positioning data, and gradually carry out precise positioning. This method first treats the Earth as a two-dimensional coordinate system and obtains two-dimensional information of smartphones through GPS positioning. This information is expressed in the form of longitude and latitude, and the direction of the smartphone in the two-dimensional coordinate system is obtained through a magnetic field sensor. Finally, the tilt angle of the smartphone is obtained grounded on the acceleration sensor. Through the above process, the precise positioning of smartphones is gradually derived. In order to achieve the above conversion, the change in tilt angle of the smartphone in the coordinate system needs to be calculated.

Assuming a two-dimensional coordinate system composed of longitude and latitude is obtained through GPS positioning, in which points $R\left( {a_{1} ,b_{1} } \right)$, $a_{1}$ and $b_{1}$ are set to represent longitude and latitude. Convert the 2D coordinates of the point to the XZ plane of the virtual 3D coordinate system, and set the coordinates to $\left( {x_{1} ,z_{1} } \right)$. Through GPS positioning, the longitude and latitude of this point can be obtained in real-time, denoted as $\left( {a_{n} ,b_{n} } \right)$. The corresponding three-dimensional XZ coordinate is $\left( {x_{n} ,z_{n} } \right)$, and the conversion coefficient $K$ of the two coordinate systems can be obtained, which is expressed as Eq. (7) ^17^.

7 $$\left\{ \begin{gathered} K_{x} = \frac{{x_{n} - x_{1} }}{{a_{n} - a_{1} }} \hfill \\ K_{z} = \frac{{z_{n} - z_{1} }}{{b_{n} - b_{1} }} \hfill \\ \end{gathered} \right.$$

In Eq. (7), $K_{x}$ is the conversion ratio coefficient of longitude to the X axis in the 3D coordinate system, and $K_{z}$ is the conversion ratio coefficient of latitude to the Z axis in the 3D coordinate system. From this, the Eq. (8) for the three-dimensional coordinate $\left( {x_{n} ,z_{n} } \right)$ of the point can be derived.

8 $$\left\{ \begin{gathered} x_{n} = \left( {a_{n} - a_{1} } \right) \times K_{x} + x_{1} \hfill \\ z_{n} = \left( {b_{n} - b_{1} } \right) \times K_{z} + z_{1} \hfill \\ \end{gathered} \right.$$

Equation (8) can obtain real-time position information of the XZ plane of the smartphone in the virtual three-dimensional coordinate system. Next, the tilt angle of the smartphone can be calculated. Assuming that in a virtual three-dimensional space, a smartphone tilts at a certain angle, and gravity generates three components in three coordinate axes: $G_{x}$, $G_{y}$, and $G_{z}$. At this point, the corresponding acceleration components generated are $A_{x}$, $A_{y}$, and $A_{z}$, and the calculation Eq. (9) for the tilt angle $r$ between the x-axis and the horizontal axis can be obtained.

9 $$r = \arctan \frac{{A_{x} - G_{x} }}{{A_{y} - G_{y} }}$$

The angle change of the tilt angle $r$ obtained in Eq. (9) can determine the real-time status of the smartphone. When $r \in \left( { - 45,45} \right),\left( {135,225} \right)$, the phone is in vertical screen mode; when $r \in \left[ { - 90, - 45} \right],\left[ {45,135} \right],\left[ {225,270} \right]$, the phone is in landscape mode. When the phone is in vertical screen mode, the calculation formula for the pitch angle $\theta$ between the Y-axis and Z-axis in the three-dimensional coordinate system can be made as Eq. (10).

10 $$\theta = \left\{ \begin{gathered} \arctan \frac{{A_{z} - G_{z} }}{{A_{y} - G_{y} }}\left( {r \in \left( { - 45,45} \right)} \right) \hfill \\ - \arctan \frac{{A_{z} - G_{z} }}{{A_{y} - G_{y} }}\left( {r \in \left( {135,255} \right)} \right) \hfill \\ \end{gathered} \right.$$

Equation (10) can calculate the pitch angle of the YZ plane under different tilt angles in vertical screen mode. Similarly, the calculation formula for the pitch angle $\theta$ between the X-axis and Z-axis in the 3D coordinate system in landscape mode can be obtained as Eq. (11).

11 $$\theta = \left\{ \begin{gathered} \arctan \frac{{A_{z} - G_{z} }}{{A_{x} - G_{x} }}\left( {r \in \left( {45,135} \right)} \right) \hfill \\ - \arctan \frac{{A_{z} - G_{z} }}{{A_{x} - G_{x} }}\left( {r \in \left[ { - 90, - 45} \right],\left[ {225,270} \right]} \right) \hfill \\ \end{gathered} \right.$$

According to Eq. (11), the pitch angle of the XZ plane at different tilt angles can be obtained in landscape mode. The above are the tilt angles of smartphones in different modes. After calculating the angle value, it can be substituted into the actual orientation map of the smartphone for analysis.

**Figure 4:**
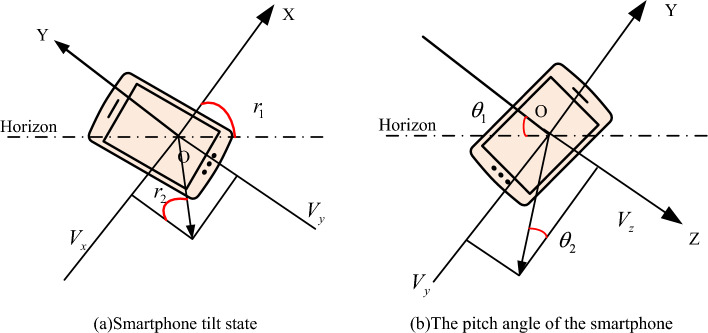
Tilt angle and pitch angle of the smartphone.

Figure 4 shows the tilt and pitch angles of the smartphone. Figure 4a shows the tilt state of the smartphone, where $V_{x}$ represents the resultant force component generated on the X-axis and $V_{y}$ represents the resultant force component generated on the X-axis. $r_{1}$ and $r_{2}$ are both inclined angles, and the calculation formula is Eq. (10). In line with the triangle theorem, it is known that the two angles are equal. Figure 4b shows the pitch angle of the smartphone. $\theta_{1}$ is the pitch angle in the vertical state, and $\theta_{2}$ is the pitch angle in the horizontal screen state. The calculation formula is shown in Eq. (11).

**Figure 5:**
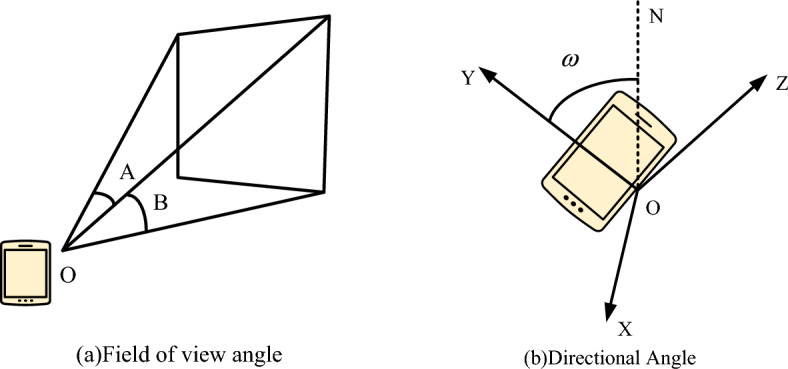
Field of view and orientation angle of smartphones.

Figure 5 shows the field of view and direction angle of a smartphone. Figure 4a shows the field of view angle, where angle A represents the angle between the left and right directions of the field of view angle, and angle B represents the angle between the upper and lower directions of the field of view angle. Due to the degree of the field of view angle affecting the visual range of the virtual perspective, to improve the matching with the real scene, the degree of the virtual field of view angle and the mobile phone field of view angle need to be consistent. Figure 4b shows the directional angle, which is the angle $w$ generated between the Y-axis and the due north direction when the smartphone rotates around the Z-axis in the virtual three-dimensional coordinate system. The value of $w$ represents the orientation of the smartphone. When $w$ is 0, the orientation is due north. When $w$ is 90, the orientation at this time is due east. When $w$ is 180, the orientation at this time is due south. When $w$ is 270, the orientation at this time is due west.

**Figure 6:**
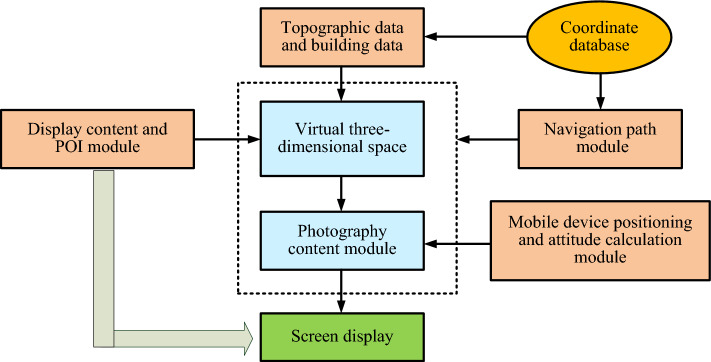
Flowchart of augmented reality navigation.

Combined with the above research, the flow chart of AR navigation in Fig. 6 can be obtained by integrating the relationship between IS-tech and virtual space transformation. In Fig. 6, the AR navigation system consists of multiple functional modules that work together. As shown in the figure, the terrain scene module is the fundamental module of the navigation system. This module mainly analyzes terrain scenes using image segmentation methods, and then uses sensor positioning systems to locate intelligent devices, transforming them into virtual three-dimensional space for expression. Eventually, it is reflected on smart screens, providing users with AR navigation functions that match real-world scenarios.

## Analysis of AR navigation results based on image segmentation and sensor T & R-technology

### Performance analysis of GA-OTSU-Canny segmentation algorithm

The mobile AR navigation system has been applied in real life and its performance has been verified. The residential area was selected as the experimental site, which includes landmarks, forks, living and entertainment venues, buildings and other landmarks, and is densely distributed. Firstly, the navigation system will be developed on the platforms Android Studio and Unity, and then the system will be loaded into the smartphone client. Later, the AR navigation system will be used for practical application at the experimental site. In order to test the performance of the GA-OTSU-Canny algorithm in the image segmentation module, different algorithms will be cited in the system for comparison and analysis during software development.

**Figure 7:**
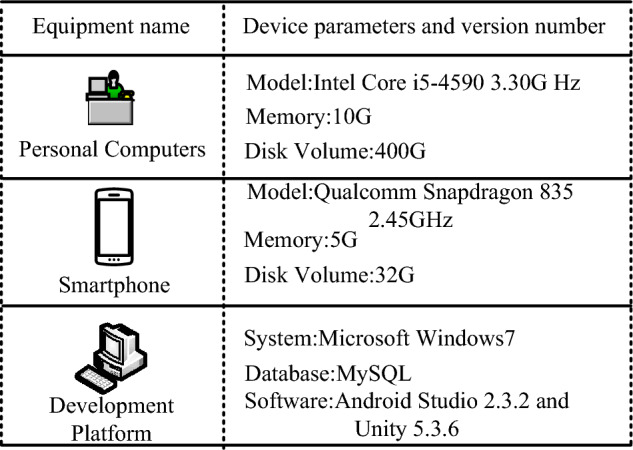
Device parameters and software versions.

Figure 7 shows the device parameters and software versions used during the experiment. To test the performance of the GA-OTSU-Canny algorithm in the image segmentation module, two other common image segmentation algorithms: The Minimum Cross-entropy Threshold algorithm (MCET) and the adaptive threshold algorithm, need to be referenced in the navigation system during software development. The algorithm validation dataset used in this study is the Cambridge-driving Labeled Video Database, which was acquired and produced by the Vision Group at the University of Cambridge. There are 32 semantic sub-categories and 11 semantic de-categories in the Cambridge-driving Labeled Video Database. The dataset contains street images of roads in various weather conditions and at different time points, which can be perfectly applied to the validation of the image segmentation algorithm in the paper.

The above navigation system is experimented with three different image segmentation algorithms, and the good performance of GA-OTSU-Canny is verified by analyzing the iterations of different algorithms, the loss curves, the edge segmentation time and edge segmentation accuracy, the localization deviation and the localization accuracy.

**Figure 8:**
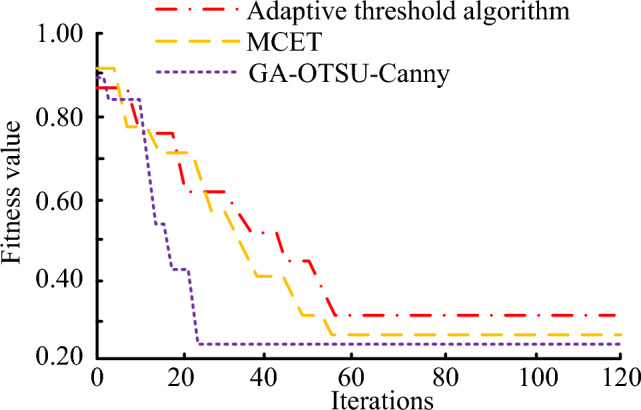
Iteration curves for different algorithms.

The iteration curves of different image segmentation algorithms are shown in Fig. 8. From Fig. 8, it can be seen that with the increase of the number of iterations, the GA-OTSU-Canny algorithm can iterate to the stable adaptation value the fastest. When it is iterated to 23 times, the best adaptation value of the algorithm is 0.22. Compared with the other two segmentation algorithms, GA-OTSU-Canny can be iterated to a stable state faster, so the stability of the algorithm is better.

**Figure 9:**
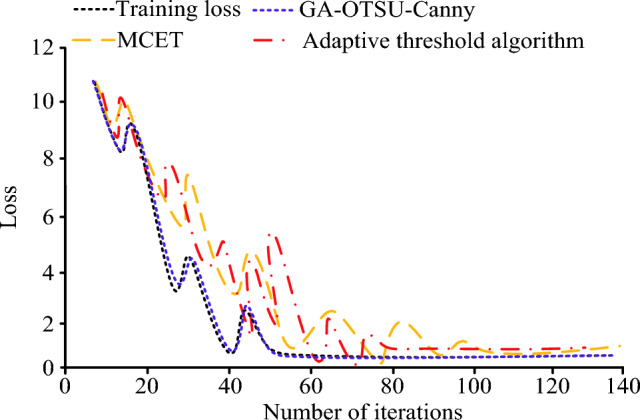
Loss curves for different algorithms.

The variation of the actual loss graph for different algorithms is shown in Fig. 9. Compared to the other two image segmentation algorithms, the actual loss curve of the GA-OTSU-Canny algorithm and the training loss curve can be highly overlapped, thus indicating that the algorithm has a smaller loss error in the actual testing process and is able to perform better image segmentation.

**Figure 10:**
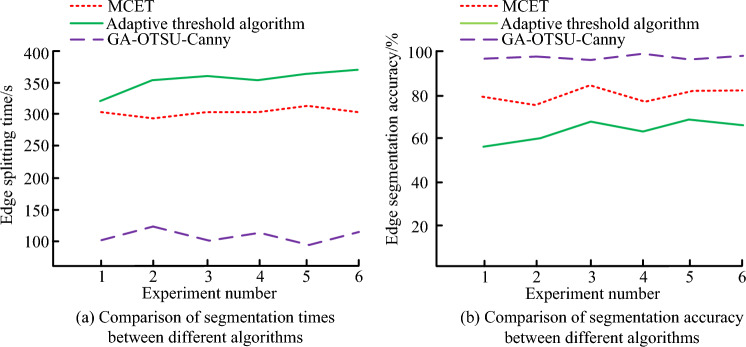
Edge segmentation time and accuracy values under different algorithms.

Figure 10 shows the comparison of edge segmentation time and edge segmentation accuracy using adaptive threshold algorithm, MCET and GA-OTSU-Canny algorithms. The efficiency of edge segmentation time determines the edge segmentation performance of the algorithm, and the accuracy of edge segmentation reflects the degree of superiority of the algorithm in processing images. For performance testing of edge segmentation time and segmentation accuracy, a total of 6 parallel experiments were conducted using different image samples as independent variables. In Fig. 10a, the adaptive threshold algorithm requires the most edge segmentation time, followed by MCET and GA-OTSU-Canny. The time required for edge segmentation using GA-OTSU-Canny is much lower than the other two algorithms, indicating higher efficiency. In Fig. 10b, the maximum edge segmentation accuracy values of the adaptive threshold algorithm, MCET, and GA-OTSU-Canny edge segmentation algorithms are 70.3%, 89.5%, and 99.5%, respectively. This indicates that the GA-OTSU-Canny algorithm performs the best in image edge segmentation. In summary, the GA-OTSU-Canny algorithm has the best edge segmentation performance among the three algorithms.

**Figure 11:**
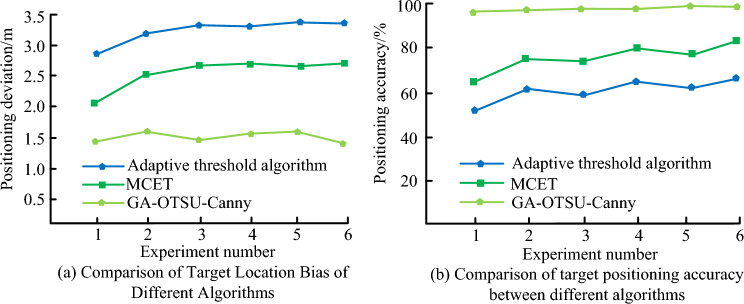
Comparison of target positioning deviation and positioning accuracy under different algorithms.

Figure 11 shows the positioning deviation and accuracy of the adaptive threshold algorithm, MCET, and GA-OTSU-Canny algorithms for target localization. The size of positioning deviation and positioning accuracy values is the key to determining the accuracy of a navigation system. Set up six parallel experiments with different target positioning points as independent variables. In Fig. 11a, the positioning deviation under the GA-OTSU-Canny algorithm is smaller compared to the other two algorithms. The maximum positioning deviations of GA-OTSU-Canny, adaptive threshold algorithm, and MCET are 1.6 m, 3.3 m, and 2.5 m, respectively. In Fig. 11b, the maximum positioning accuracy values of the adaptive threshold algorithm, MCET, and GA-OTSU-Canny edge segmentation algorithms are 63.2%, 82.1%, and 99.2%, respectively. Therefore, the GA-OTSU-Canny algorithm has better positioning accuracy values. In summary, under the GA-OTSU-Canny algorithm, the positioning deviation of the navigation system is smaller; the positioning accuracy is higher; and the positioning effect is better.

**Figure 12:**
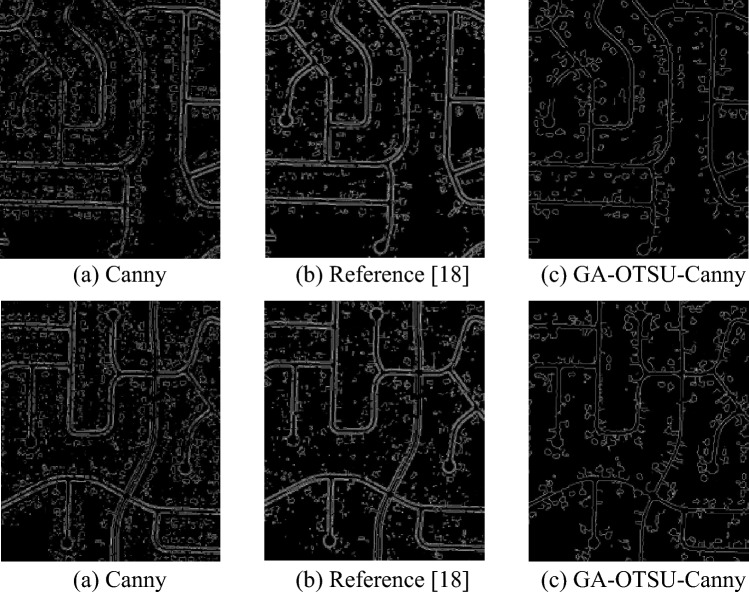
The segmentation effect of road image under different methods.

Figure 12 shows the segmentation effect of traditional Canny, reference^18^ and GA-OTSU-Canny algorithms on road images. As can be seen from Fig. 12, the road image processed by GA-OTSU-Canny algorithm has better anti-noise performance, and there are only a few pseudo-edges in the detected road edge results, and the overall edge segmentation effect is the best. However, the fracture phenomenon of road edge extracted by traditional Canny edge detection algorithm is very serious, and there are a large number of pseudo-edges caused by building, vegetation, shadow and other noise, the overall extraction effect is poor, the edge detection accuracy is low, and the noise resistance is not good. The extraction method adopted in reference^18^ is superior to the traditional Canny algorithm. The road edge information extracted by it has higher accuracy, and the road is continuous and smooth. Unlike the traditional Canny algorithm, there are fractures everywhere, but the overall segmentation effect is not as good as GA-OTSU-Canny.

### Analysis of AR navigation effects

After performance testing of the image segmentation algorithm, it was incorporated into the AR navigation system for real-life navigation experiments. The tracking and registration speed and accuracy of sensors determine the overall navigation performance. There are many factors that affect the accuracy of navigation on real roads. To achieve a high degree of overlap between virtual information and real road conditions, it is necessary to evaluate the performance of the overall navigation system from different aspects. This study analyzed the application effects of navigation systems using different algorithms in real-life scenarios.

**Figure 13:**
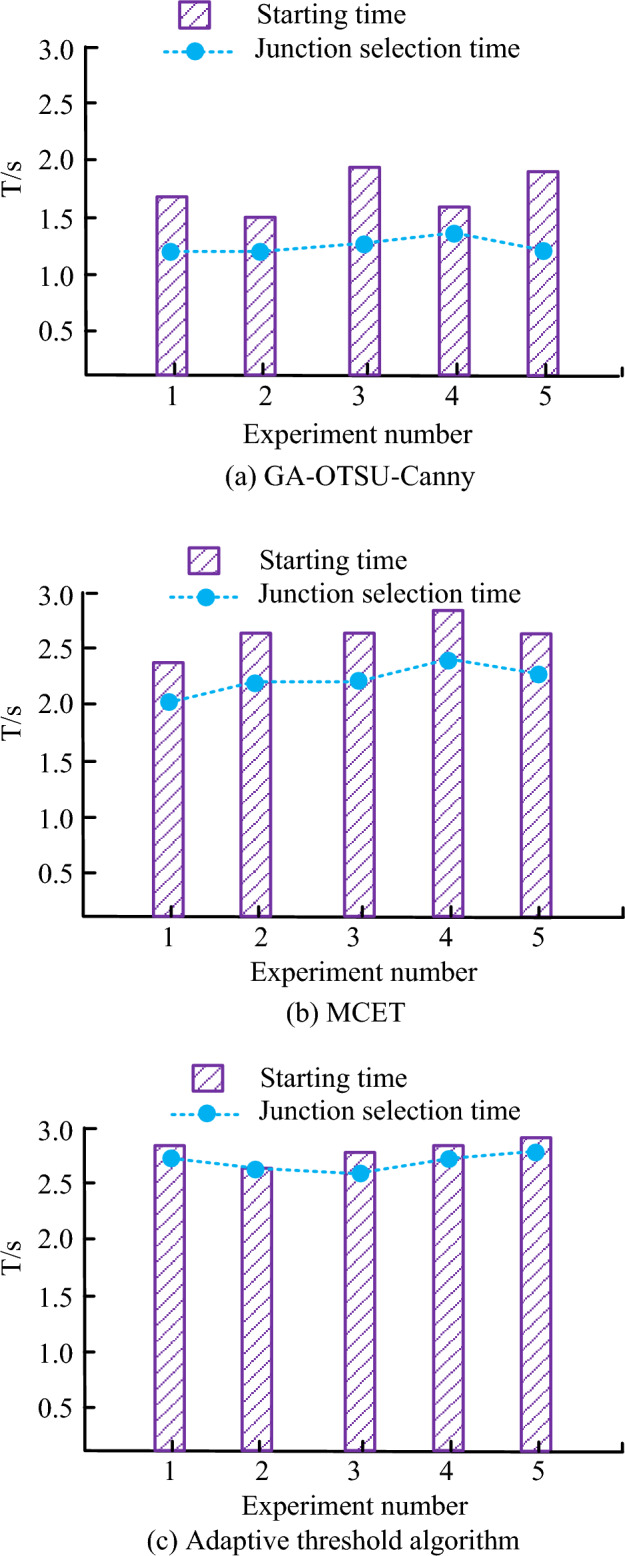
Start time and intersection selection time under different algorithms.

Figure 13 shows the comparison of the starting time and intersection selection time of the adaptive threshold algorithm, MCET, and GA-OTSU-Canny algorithms in real-time navigation. The algorithms used in Fig. 13a–c are GA-OTSU-Canny, MCET, and adaptive threshold algorithms, respectively. In five parallel experiments, the starting speed under GA-OTSU-Canny was faster than the other two algorithms, with a maximum starting time of 1.8 s and an average starting time of 1.6 s. Furthermore, the navigation system under GA-OTSU-Canny has a more agile judgment speed for selecting intersections. Figure 13 shows that the fastest intersection selection time under the GA-OTSU-Canny algorithm is only 1.2 s, which is higher than the 2.0 s speed of MCET and the 2.5 s speed of the adaptive threshold algorithm. In conclusion, the AR navigation system under GA-OTSU-Canny has a faster starting speed and a faster ability to select intersections. However, an AR navigation system with complete performance not only needs to have the above two elements, but also needs to judge the overall navigation accuracy.

**Figure 14:**
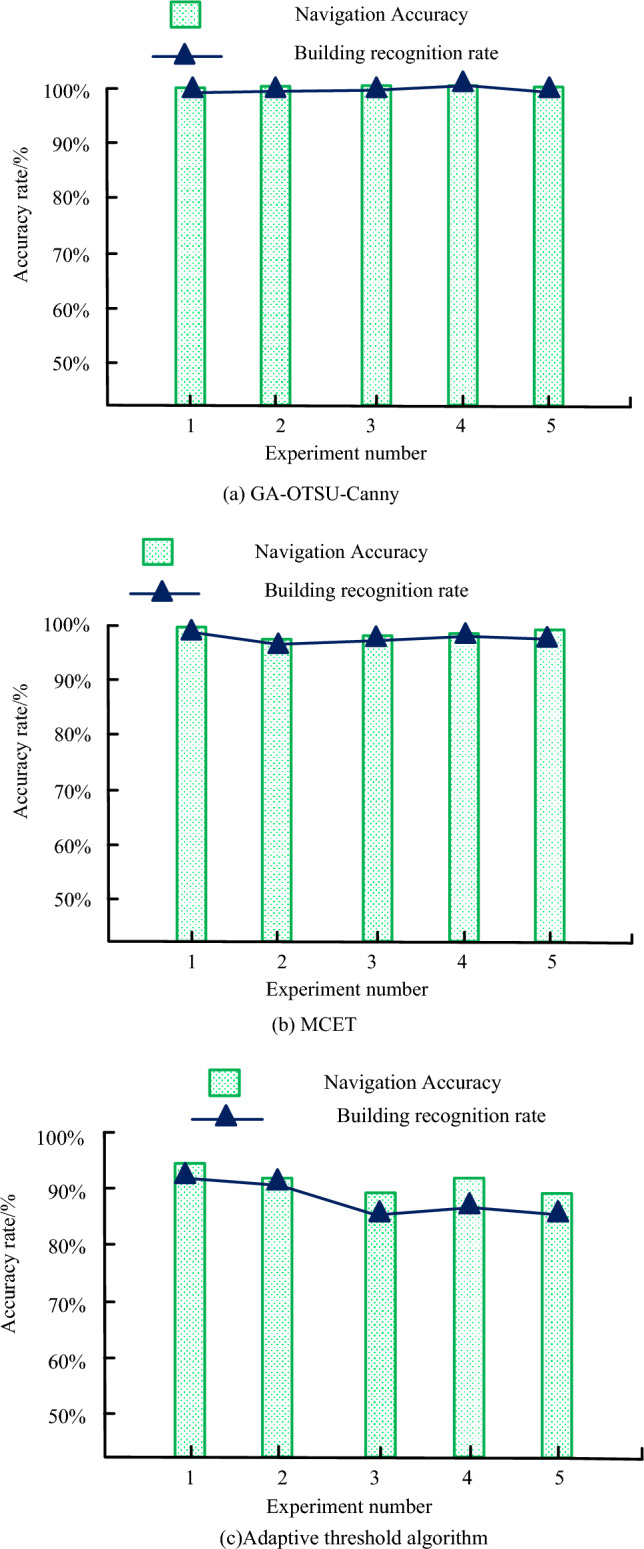
Accuracy under three algorithms.

Figure 14 shows the comparison of building recognition accuracy and navigation accuracy under three algorithms: adaptive threshold algorithm, MCET, and GA-OTSU-Canny. The algorithms used in Fig. 14a–c correspond to GA-OTSU-Canny, MCET, and adaptive threshold algorithms. In five experiments, the navigation accuracy under GA-OTSU-Canny was 100%, and the recognition accuracy of buildings in the navigation route was all above 99%. The highest recognition accuracy was 100%, which was higher than the other two algorithms. In summary, AR navigation using GA-OTSU-Canny algorithm can more accurately identify landmark buildings and guide users to the correct route.

In order to further demonstrate the performance of the navigation system incorporating the image segmentation algorithm GA-OTSU-Canny and sensor tracking alignment technology in practical applications, a rural road and an urban road were chosen for testing. The rural road is not blocked by many buildings, but its road condition is poor. The city road has better road conditions, but there are a lot of buildings blocking the road, so the image recognition accuracy of the navigation system has a higher test.

Table 1 shows the actual index test results of two different types of road conditions under the traditional navigation system and augmented reality navigation system. The performance comparison between conventional navigation and augmented reality navigation is shown in Table 1. Two different application scenarios were selected for the study to evaluate the performance of the navigation system. Navigation time, navigation accuracy, and navigation deviation were selected as the evaluation indexes of system performance. As shown in Table 1, the navigation time of the conventional GPS navigation system in urban asphalt roads is 3.2 min, the navigation accuracy is 0.75, and the navigation deviation is (8.4°, ± 4.3 m). The navigation time of the augmented reality navigation system in the urban asphalt road was 1.8 min; the navigation accuracy was improved to 0.95; and the navigation deviation was only (1.6°, ± 0.7 m). In addition, the study also compared the effectiveness of the two navigation systems in rural dirt roads. Compared with urban areas, the rural landscape is more complex and primitive, and there are many unexplored roads, so the navigation accuracy of the navigation system in rural areas is lower than that in urban areas. In particular, the navigation time of traditional GPS navigation system on rural dirt roads is 5.6 min, the navigation accuracy is 0.69, and the navigation deviation is (12.5°, ± 7.3 m). The navigation time of the augmented reality navigation system on a wide urban asphalt road was 2.4 min, the navigation accuracy improved to 0.91, and the navigation deviation was only (3.1°, ± 1.8 m).

**Table 1:** Performance comparison of traditional navigation and augmented reality navigation.

Navigation system	Application environment	Navigation time	Navigation accuracy	Navigation deviation
Traditional GPS navigation	Urban asphalt roads	3.2 min	0.75	(8.4°, ± 4.3 m)
	Rural dirt roads	5.6 min	0.69	(12.5°, ± 7.3 m)
Augmented reality navigation	Urban asphalt roads	1.8 min	0.95	(1.6°, ± 0.7 m)
	Rural dirt roads	2.4 min	0.91	(3.1°, ± 1.8 m)

In summary, the two navigation systems showed different navigation time, navigation accuracy and navigation deviation under different road conditions. Compared with urban road conditions, rural roads have poorer performance in all navigation indexes due to the presence of large human interference factors in rural roads. Most of the rural roads are considered dirt roads, and although there are no more obstacles to block them, there are more minor road branches, thus reducing the overall navigation accuracy of the navigation system.

The navigation path from the gate of a university to the dormitory of girls' building A is selected as an experimental object, and three different image segmentation processing algorithms, namely Convolutional Neural Network (CNN), Whale Optimization Algorithm (WOA), and Differential Optimization-Sparrow Search Algorithm (DE-SSA), are introduced as comparative algorithms. Evolution-Sparrow Search Algorithm (DE-SSA) three different image segmentation processing algorithms as comparison algorithms. The three road image segmentation algorithms are combined with traditional navigation technology and virtual reality augmented navigation technology respectively, and the final actual navigation effects of various navigation methods are obtained as shown in Table 2. In Table 2, the navigation times of Method 1, Method 2, Method 3, Method 4, Method 5, Method 6 and the methods proposed in the paper are 23.64 min, 19.93 min, 18.62 min, 14.91 min, 15.05 min, 11.28 min and 5.36 min, respectively, and the navigation accuracy rates are 81.73%, 87.69%, 88.56%, 91.32%, 92.08%, 95.22% and 98.96%, respectively.

**Table 2:** Actual navigation effects of different navigation methods.

Methods	Navigation time	Navigation accuracy
CNN + traditional navigation ( notation method 1)	23.64 min	81.73%
CNN + virtual reality augmented navigation (notation 2)	19.93 min	87.69%
WOA + traditional navigation (notation 3)	18.62 min	88.56%
WOA + virtual reality augmented navigation (notation 4)	14.91 min	91.32%
DE-SSA + conventional navigation (notation 5)	15.05 min	92.08%
DE-SSA + virtual reality augmented navigation (notation 6)	11.28 min	95.22%
GA-OTSU-Canny + ^21^	6.05 min	97.53%
Methods mentioned in the text	5.36 min	98.96%

Further, two of the latest image segmentation algorithms are selected and combined with different navigation frameworks for comparative testing, and the test results of several methods are shown in Table 3. In Table 3, when literature^19^ and literature^20^ combine virtual reality augmented navigation technology, their navigation time and navigation accuracy can reach their highest levels respectively, with navigation time of 7.78 min and 7.46 min, and navigation accuracy of 94.59% and 96.45%, respectively. By combining the GA-OTSU-Canny image segmentation method proposed in the paper with the literature^21^, it can be found that the optimized SLAM navigation technology can effectively shorten the navigation time and improve the navigation accuracy rate, making the navigation time as low as 6.05 min and the navigation accuracy rate as high as 97.53%. According to Tables 2 and 3, the proposed method in this study still has the best performance and can achieve the shortest navigation time and the highest navigation accuracy.

**Table 3:** Comparison of navigation performance of different segmentation algorithms combined with navigation framework.

Methods	Navigation time	Navigation accuracy
^19^ + traditional navigation	8.96 min	92.32%
^19^ + virtual reality augmented navigation	7.78 min	94.59%
^19^ + SLAM	8.15 min	93.72%
^20^ + traditional navigation	8.02 min	95.10%
^20^ + virtual reality augmented navigation	7.46 min	96.45%
^20^ + SLAM	7.83 min	96.08%
Methods mentioned in the text	5.36 min	98.96%

**Figure 15:**
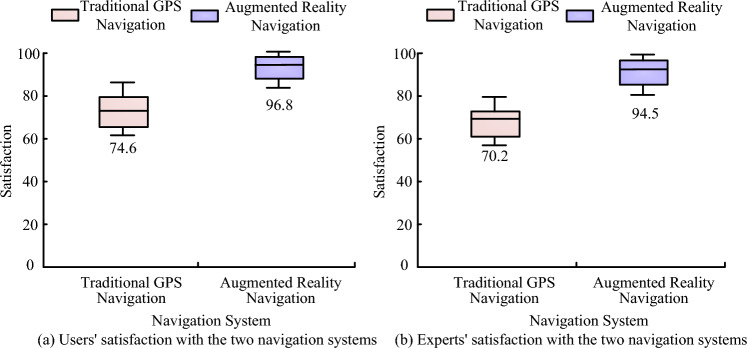
Satisfaction of experts and users with both navigation systems.

Figure 15 shows the satisfaction of experts and users with the two navigation systems. A score of 100 is set as the best satisfaction, when the higher the scores of customers and experts, the higher their satisfaction. Figure 15a shows the user satisfaction with the conventional GPS navigation system and the augmented reality navigation system. Figure 15b shows the experts' satisfaction with the traditional GPS navigation system and the augmented reality navigation system. The combined results in Fig. 15a,b show that users and experts are 74.6 and 70.2 satisfied with the traditional GPS navigation system and 96.8 and 94.5 satisfied with the traditional augmented reality navigation system, respectively, which shows that both experts and users believe that augmented reality navigation system can bring better navigation experience and therefore can obtain higher satisfaction scores.

## Discussion

With the development of computer technology, deep learning and image processing techniques, various image algorithms are gradually being applied in various fields. Liu et al. combined machine vision technology, immune biosensors, and signal amplification biochips to build an intelligent diagnostic system for the detection of bacteria, aiming to detect urinary tract infections through the detection of bacteria. The application results showed that the marked image signals were well captured by machine vision algorithms as a way to provide better dynamic detection results^22^. In order to minimize the damage of natural disasters, more and more researchers are using intelligent algorithms and emerging technologies for natural disaster prediction. Nair et al. proposed a deep learning based machine vision algorithm and used it for flood depth measurement and estimation. An image segmentation algorithm based on fuzzy logic and image color based image segmentation was also proposed with the aim of predicting the flood extent from various flood images. Experimental results showed that the prediction accuracy of this algorithm was as high as 83.1%^23^. Zhang et al. proposed a knitting needle cylinder fault detection system incorporating laser detection and machine vision algorithm in order to reduce the defective products produced by sock machines due to knitting needle cylinder faults. During the operation of the system, the laser signal is collected using a photodetector, and the machine is stopped immediately when a fault signal is detected, and the image of the faulty machine is transmitted back and fault identification is performed. The performance of the system was examined and found to have a classification time of only 0.002 s for faulty images^24^. Liu et al. developed an intelligent sorting system based on machine vision using neural network algorithms. Firstly, a camera was used to acquire the image of the item to be sorted, then RBF neural network algorithm was used to acquire the defective features in the image and localize the features, and finally an air jet was designed to extrude the defective items from the conveyor belt. The performance of the whole system was tested and it was found that the system has a high removal rate of 91.7% for defective items^25^. To facilitate port automation, Miao et al. proposed a 3D point cloud hull modeling and operational target recognition algorithm based on a laser measurement collection system. In the recognition algorithm, a real-time point cloud is projected into the coordinate system and the 3D point cloud is converted into a 2D image for the purpose of fast identification of operational targets. The recognition algorithm was tested and found to have a high recognition accuracy and to be able to recognize the operational targets in real time^26,27^. As shown above, there have been a number of studies on image segmentation and image recognition algorithms. All the above-mentioned types of image algorithms have achieved good performance and application results. To address a series of problems such as low navigation accuracy and poor recognition of navigation targets that exist in current navigation applications, this study innovatively combines image segmentation algorithms with multi-sensor technology and uses both to design an augmented reality navigation system.

In augmented reality navigation, the combination of image segmentation and sensor tracking and registration technologies opens up new application possibilities^28^. The linkage of the two not only increases the accuracy of navigation but also enhances the user experience. Image segmentation is a method for extracting useful information from images, and the method is crucial for augmented reality navigation because it allows virtual objects to fit naturally into the user's actual environment^29^. Over the past few years, numerous researchers have invested in this field and have used a range of deep learning methods to segment images. However, despite some progress in problems such as semantic segmentation and instance segmentation, there are still some challenges in image segmentation techniques, and one of the main challenges is image segmentation in complex environments. For example, the accuracy of image segmentation can be compromised in the presence of lighting changes, occlusions, or complex backgrounds. In addition, most of the existing image segmentation algorithms require high hardware and computational resources, which also leads to the fact that it may be impractical to apply them to augmented reality navigation devices. On the other hand, sensor tracking and registration technology is the key to connect the virtual world with the real world. Sensor tracking registration techniques typically use the built-in sensors of smartphones or other mobile devices for tracking and localization of device motion. However, the accuracy of these sensors is affected by many factors, such as environmental interference and the device's own errors. Therefore, improving the accuracy and robustness of sensor tracking is an important current research direction. Considering the above-mentioned points, this research designs an augmented reality navigation system using GA-OTSU-Canny image segmentation algorithm with multi-sensor technology. The results show that the adopted image segmentation algorithm can recognize building images in complex environments well, and the designed augmented reality navigation system has better navigation performance than the traditional sensor navigation system, which can improve the user satisfaction and usage to a greater extent.

## Conclusion

Navigation has become a necessary tool for modern people's travel, and users can obtain navigation routes by reading virtual information from smart screens. To combine virtual information in navigation systems with people's real life scenarios and improve the efficiency of users' access to route information, this study used GA-OTSU-Canny algorithm and sensor technology in image segmentation to analyze the application of AR navigation. The experimental results show that GA-OTSU-Canny has faster efficiency in segmenting image edges. The GA-OTSU-Canny algorithm is applied to the AR system for real-time navigation. The average starting time of the navigation system under this algorithm is 1.6 s, and the intersection can be selected within 1.2 s at the fastest. On this efficient basis, the navigation route accuracy of the system reaches 100%, and the recognition rate of all buildings in the route is above 99%. In conclusion, this technology has certain feasibility. This experiment combines image processing algorithm and sensor technology to build an augmented reality navigation system, which not only achieves better image segmentation effect but also improves the accuracy of navigation. However, since this experiment only studied the image recognition technology and multi-sensor technology in the augmented reality navigation system, there is still some room for research. Since the navigation system may have different navigation accuracy in different road conditions, more road conditions should be selected for analysis in subsequent studies.

## Future work

This study combines image segmentation algorithms with multi-sensor technology and designs an augmented reality navigation system. Analysis of the algorithm performance and navigation system performance reveals that the image segmentation algorithm used has a better performance and the designed augmented reality navigation system outperforms the traditional sensor navigation system. Although image segmentation and sensor tracking registration techniques have great potential for augmented reality navigation, there are still many issues that need to be addressed. Considering these issues, future research directions can be developed from the following perspectives. First, research on more efficient and accurate image segmentation algorithms, including the handling of different environmental conditions, such as illumination and occlusion, as well as the optimization of computational efficiency. Second, better sensor fusion and calibration methods are investigated to improve the accuracy and robustness of sensor tracking. This may require combining different types of sensors, such as optical, magnetic, and inertial sensors, to obtain more accurate tracking results. Finally, better interaction and display methods for practical augmented reality navigation applications are investigated to enhance the user experience to a greater extent. Overall, future research needs to delve deeper into these issues and develop more efficient, accurate, and adaptive techniques to advance augmented reality navigation technology.

## Data Availability

The datasets used and/or analysed during the current study available from the corresponding author on reasonable request.
